# Systematic Characterization of the Clinical Relevance of KPNA4 in Pancreatic Ductal Adenocarcinoma

**DOI:** 10.3389/fonc.2022.834728

**Published:** 2022-03-29

**Authors:** Jingpiao Bao, Chaoliang Xu, Bin Li, Zengkai Wu, Jie Shen, Pengli Song, Qi Peng, Guoyong Hu

**Affiliations:** ^1^ Department of Gastroenterology, Shanghai General Hospital, Shanghai Jiao Tong University School of Medicine, Shanghai, China; ^2^ Shanghai Key Laboratory of Pancreatic Disease, Institute of Pancreatic Disease, Shanghai Jiao Tong University School of Medicine, Shanghai, China; ^3^ Laboratory of Cancer Genomics and Biology, Department of Urology, Shanghai General Hospital, Shanghai Jiao Tong University School of Medicine, Shanghai, China

**Keywords:** karyopherin subunit alpha 4, pancreatic ductal adenocarcinoma, prognostic biomarker, tumor microenvironment, FAK signaling

## Abstract

**Background:**

Pancreatic ductal adenocarcinoma (PDAC) is one of the most lethal malignancies with poor prognosis. Karyopherin subunit alpha 4 (KPNA4) is a nuclear transport factor and plays tumor-promoting roles in multiple cancers. However, the roles of KPNA4 in PDAC still remain unknown. This study investigated the prognostic value of KPNA4 and its potential functions in PDAC and tumor microenvironment.

**Methods:**

LinkedOmics was utilized to screen genes with survival significance in PDAC. KPNA4 expression was analyzed using multiple datasets and verified in PDAC cells and clinical samples by qRT-PCR and immunohistochemistry. Clinical correlation and survival analyses were conducted to identify the clinical significance and prognostic value of KPNA4 in PDAC patients. Subsequently, *KPNA4* was knocked down in PDAC cell lines, and CCK-8, colony formation and wound healing assays were performed to test the functions of KPNA4 *in vitro*. Immune infiltration analysis was performed to explore the potential roles of KPNA4 in the tumor microenvironment of PDAC. Moreover, functional analyses were conducted to explore the underlying mechanism of KPNA4 in the progression of PDAC.

**Results:**

We found KPNA4 was significantly upregulated in PDAC cells and tissues. KPNA4 expression was associated with tumor progression in PDAC patients. Survival analyses further revealed that KPNA4 could act as an independent predictor of unfavorable survival for PDAC patients. *KPNA4* knockdown suppressed the viability, colony formation and migration of PDAC cells. Moreover, KPNA4 was correlated with immunosuppressive cells infiltration and T cell exhaustion in the tumor microenvironment of PDAC. Finally, functional analyses indicated the association of KPNA4 with focal adhesion kinase (FAK) signaling, and *KPNA4* silencing significantly decreased the expression of *FAK* and *PD-L1*.

**Conclusions:**

This study revealed that KPNA4 is an independent prognostic biomarker for PDAC and plays a tumor-promoting role by facilitating proliferation and migration of cancer cells and participating in immune infiltration, which may be mediated by FAK signaling and PD-L1 expression. These results provide a novel and potential therapeutic target for pancreatic cancer.

## Introduction

In the United States, pancreatic cancer is predicted to be the second most common cause of cancer death by 2030, with the lowest 5-year survival rate among common solid cancers (1, 2). Also in China, the 5-year survival rate of pancreatic cancer is only 9.9%, which has not improved obviously over the past decade, and is expected to continue to rise (3). Pancreatic ductal adenocarcinoma (PDAC) is the most common type of pancreatic cancer, and more than half of patients are diagnosed at an advanced stage and have systemic metastases, with only 15-20% of patients have the chance of surgical resection (2, 4). At present, the management of PDAC in China is still mainly based on surgery, chemotherapy and radiotherapy, supplemented by interventional therapy, best supportive care and traditional Chinese medicine (5). Despite the considerable improvements in surgical therapy, PDAC still carries a dismal prognosis (4). Also, conventional cytotoxic chemotherapy has an extremely limited effect on the prognosis (6). Therefore, it is necessary to further explore the occurrence and development mechanisms of PDAC and discover highly effective prognostic biomarkers, so as to improve the disease prediction and therapy decision of PDAC.

Desmoplasia is a key histopathological feature of PDAC, manifested by extensive fibrosis surrounding primary and metastatic tumor sites (7). The highly fibrotic stroma can act as a mechanical barrier for tumor cells, thus creating a tumor microenvironment (TME) conducive to tumor growth, where therapeutic drugs are difficult to reach and immune cells exhibit a poor infiltration (8). In addition, PDAC is recognized as an immunologically cold tumor type (9). These may explain why PDAC patients are lowly responsive to immune checkpoint inhibitors (10). Therefore, further research on PDAC TME is urgently needed to find ways to maximize the tumoricidal activity of immune cells, thus enhancing the anti-tumor response.

Karyopherin subunit alpha 4 (KPNA4), which is a member of the nuclear transport factors KPNA family, has been reported to be associated with multiple cancers, including prostate cancer, hepatocellular carcinoma, lung cancer, ovarian cancer, glioblastoma and other malignancies. These studies reveal that KPNA4 exerts a tumor-promoting role by facilitating cell cycle progression, epithelial-mesenchymal transition (EMT), angiogenesis and other cancer-related processes (11–16). Moreover, *KPNA4* was found to be correlated with immune cell infiltration in hepatocellular carcinoma (16), suggesting that *KPNA4* expression may have an effect on TME and anti-tumor immunity. However, so far, the potential function of KPNA4 in PDAC and its impact on the TME still remain to be elucidated.

In this study, we aimed to analyze the expression and prognostic value of KPNA4 in PDAC and identified KPNA4 as a potential prognostic biomarker for PDAC through bioinformatics analysis. Loss-of-function experiments revealed that KPNA4 exerts a promoting role in malignant behaviors of PDAC cells. Moreover, immune infiltration analysis further revealed the potential immunosuppressive effect of KPNA4 on PDAC TME. Finally, functional analyses demonstrated that KPNA4 may be involved in focal adhesion kinase (FAK) signaling, which can promote tumor progression and metastases by acting on tumor cells and stromal cells in the tumor microenvironment (17). Our experimental data revealed the positive regulatory effect of KPNA4 on the expression of FAK and immune checkpoint molecule PD-L1. Our results are expected to provide new ideas for the prognosis prediction and targeted therapy of PDAC.

## Materials and Methods

### Identification of Genes With Survival Significance in PDAC

LinkedOmics (http://www.linkedomics.org/) is a publicly accessible online database to analyze multi-omics data from 32 kinds of TCGA human cancer (18). This database has the unique ability to display the survival significance of all human genes in specific tumor types. Thus, in our study, in order to find immune-related biomarkers with potentially high prognostic value in PDAC, LinkedOmics was utilized to obtain a list of genes with survival significance in PDAC, and these genes were ranked by their false discovery rate (FDR) value.

### Analyses of KPNA4 Expression and Its Correlation With FAK Signaling-Related Genes

Gene Expression Profiling Interactive Analysis (GEPIA) (http://gepia.cancer-pku.cn/) is a multifunctional online database for analyzing gene expression and clinical data from TCGA and the GTEx database, containing 9,736 tumors and 8,587 normal samples (19). Considering the scarcity of RNA-seq data of normal pancreas sample (e.g. only four normal samples in TCGA), GEPIA can largely compensate for this problem. GEO (http://www.ncbi.nlm.nih.gov/geo/) is a public database that collects microarray, high-throughput sequencing and other forms of data uploaded by global researchers, providing a large number of datasets for verifying the expression changes of target genes (20, 21). In the present study, GEPIA and two PDAC microarrays GSE15471 and GSE16515 obtained from the GEO database were utilized to analyze the mRNA expression of *KPNA4* in PDAC and normal samples, as well as its correlation with FAK signaling-related genes (*FAK*, *PIK3CA*, *AKT1* and *CD274*). To further enrich the dataset sources, Oncomine (http://www.oncomine.org/), which contains various tumor microarray datasets, was also used to verify the expression change of *KPNA4* in microarrays of PDAC tissues. The Human Protein Atlas (HPA) (http://www.proteinatlas.org/) is a publicly available database for human proteins mapping in cells, tissues and organs by various omics technologies, providing the expression of the target gene at the protein level (22). In this study, HPA was utilized to obtain the immunohistochemical images of KPNA4 expression in normal pancreas and PDAC tissues, then direct comparison was performed between the two groups. *p* < 0.05 was regarded to be statistically significant.

### Clinical Samples and Immunohistochemical Staining

PDAC and adjacent normal pancreas samples were surgically obtained from twenty-three patients to validate the upregulation of KPNA4 in PDAC. This work was approved by the Ethics Committee of Shanghai General Hospital (approval number: 2021KY116). The clinical characteristics of each patient are shown in **Supplementary Table 1**. Immunohistochemical staining of the paraffin-embedded tissue sections were conducted following standard protocols. The tissue sections were incubated overnight with a rabbit polyclonal antibody to human KPNA4 (Sigma-Aldrich, HPA043154). The histochemistry score was determined by multiplying the stained intensity scores by the extent of positive cells. The stain intensity was scored as follows: 0, blank; 1, yellow; 2, dark yellow; and 3, brown. The extent of positive cells was scored as follows: 0, 0-5%; 1, 6-25%; 2, 26-50%; 3, 51-75%; and 4,76-100%. All slides were independently accessed by two experienced pathologists in a blinded fashion.

### Clinical Relevance Analysis of KPNA4 in PDAC

The Cancer Genome Atlas (TCGA) (http://portal.gdc.cancer.gov/) is a landmark cancer genomics project, providing sequencing and clinical information for 33 types of human cancer (23). mRNA expression data of 177 PDAC and 4 normal pancreas samples determined by the value of Fragments Per Kilobase per Million, and incomplete clinical data of 185 PDAC patients were obtained from TCGA database. In our study, 173 PDAC samples with mRNA expression and clinical data were used to perform clinical correlation and stratified survival analysis of KPNA4. Furthermore, 120 PDAC samples with *KPNA4* expression and complete clinical information were included in Cox regression analysis. OSpaad (http://bioinfo.henu.edu.cn/PAAD/PAADList.jsp/), which contains transcriptional expression and survival data of a total of 1319 PDAC cases from TCGA, ICGC and the GEO databases (GSE28735, GSE62452 and GSE71729) (24), was used to further validate the survival significance of KPNA4 in PDAC patients. *p* value < 0.05 was considered as statistically significant.

### Cell Culture and Treatment

The human normal pancreatic ductal epithelial cell line HPDE6-C7 and PDAC cell lines (SW1990, MIA PaCa-2, BxPC-3 and PANC-1) were all obtained from Type Culture Collection of the Chinese Academy of Science (Shanghai, China). SW1990 was cultured in RPMI 1640 medium (Gibco), and HPDE6-C7, BxPC-3, MIA PaCa-2 and PANC-1 were cultured in Dulbecco’s modified Eagle’s medium (DMEM, Gibco). The medium was supplemented with 10% fetal bovine serum (FBS, Gibco) and 1% penicillin-streptomycin (Gibco). All the cells were incubated in a humidified atmosphere containing 5% CO_2_ at 37°C. The cell culture media was replaced every 2-3 days.

To determine the function of KPNA4 in PDAC cell lines, siRNA that targeted human *KPNA4* (si*KPNA4*) and its negative control (siNC) were synthesized by RiboBio (Guangzhou, China). Three different KPNA4-specific siRNA were designed for this study (si*KPNA4*-1: GCGGAACAUUUGGUUUCAATT, si*KPNA4*-2: GCAGUAAUUGAUGCCAAUCTT, si*KPNA4*-3: CCACCACCAAUGGAA ACCATT), and si*KPNA4*-3 was selected for further loss-of-function experiments because of its high inhibition efficiency. Transient transfection in these PDAC cells was carried out by using Lipofectamine 3000 (Invitrogen) according to the manufacturer’s instructions. In our preliminary experiments, MIA PaCa-2 and PANC-1 in the 6-well-plates were treated with si*KPNA4* at different concentrations (10, 50 and 100 nM) and Lipofectamine 3000 at different volumes (2, 4, 6 and 8 μL), and results showed that 100nM si*KPNA4* concentration and 4 μL Lipofectamine were the most effective and safe conditions for *KPNA4* knockdown.

### Cell Viability and Colony Formation Assays

Cell Counting Kit-8 (CCK-8) assay and colony formation assay were performed in our study to evaluate the proliferation ability of PDAC cell lines. Briefly, for CCK-8 assay, MIA PaCa-2 and PANC-1 cells after transient transfection for 48 hours were seeded into the 96-well-plates (1000 per well), and cultured for 0, 24, 48, 72 and 96 hours. Cell viability was then examined by using the CCK-8 reagent (Dojindo, Kumamoto, Japan), and the absorbance at 450 nm was determined by the microplate reader after 2 hours incubation, with six wells per group being analyzed. For colony formation assay, cells were seeded into the 6-well-plates (1000 per well) and cultured for 7-10 days until visible colonies formed. Then, the colonies were stained with crystal violet and quantified by using Image J software, with three wells per group being analyzed.

### Wound Healing Assay

Wound healing assay was carried out in this study to investigate the migration ability of PDAC cell lines. MIA PaCa-2 and PANC-1 cells after transient transfection for 48 hours were seeded into the 6-well-plates. After cells had reached confluence, a scratch was made by a 200 μL pipette tip. Cell debris was washed away with PBS, and the medium was replaced with fresh serum-free DMEM. The wound area was photographed by using a microscope every 24 hours, with three wells per group being analyzed.

### Correlation Between KPNA4 and Tumor-Infiltrating Immune Cells

Tumor IMmune Estimation Resource (TIMER) (https://cistrome.shinyapps.io/timer/) and TIMER2.0 (http://timer.cistrome.org/) are powerful tools that can evaluate the potential impact of genes of interest on immune cell infiltration in the TME of different cancer types (25). In our work, TIMER and TIMER2.0 database were used to analyze the correlation between *KPNA4* expression and various immune cells infiltration in PDAC. *p* value < 0.05 was regarded to be statistically significant.

### Functional Enrichment Analyses and Protein–Protein Interactions (PPI) Network

LinkedOmics was used to obtain the list of *KPNA4* co-expressed genes in PDAC. The 200 positively and 200 negatively related genes with the highest correlation coefficients were included in the subsequent functional enrichment analyses.

Gene Ontology (GO) analysis composed of biological process (BP), molecular function (MF) and cellular component (CC), along with Kyoto Encyclopedia of Genes and Genomes (KEGG) pathway analysis are well-known methods to predict biological functions of gene and gene clusters. To investigate the potential roles of KPNA4 in PDAC, GO and KEGG pathway analysis were carried out on *KPNA4* co-expressed genes through The Database for Annotation, Visualization, and Integrated Discovery (DAVID) (http://david.ncifcrf.gov/) (26, 27). *p* value < 0.05 were accepted as statistically significant. In addition, the “clusterProfiler” R package was used to perform Gene Set Enrichment Analysis (GSEA) and the “enrichplot” R package was utilized to visualize the result. A nominal *p*-value < 0.05 and FDR < 0.25 were considered to be a significantly enriched gene set.

*KPNA4* co-expressed genes in PDAC were uploaded to the STRING database (http://string-db.org/), then the PPI network was obtained from this web tool (28, 29). The network was further filtered with the highest confidence score (> 0.900), and the disconnected nodes were removed. Then, the hub genes of the PPI network were calculated by using CytoHubba plug-in in Cytoscape 3.8.0 software.

### Quantitative Real-Time PCR (qRT-PCR) Analysis

Total RNA of cell lines was extracted with Trizol reagent (Takara) and reverse-transcribed into cDNA using PrimeScript RT Master Mix (Takara) according to the manufacturer’s instructions. To determine the expressions of *KPNA4* and FAK signaling-related genes in PDAC cell lines at the mRNA level, qRT-PCR analysis was performed with SYBR Premix Ex Taq II (Takara) in the QuantStudio 7 Flex Real-Time PCR System (Applied Biosystems) with *GAPDH* as an internal control. Each sample was run in triplicate, and the experiments were repeated three times independently. All qRT-PCR reactions were conducted with the following settings: 95°C for 30 s; 40 cycles of 95°C for 5 s and 60°C for 34 s. The comparative CT method (2^-△△CT^) was applied to calculate the relative expression of *KPNA4*. The specific sequences of primers used are as follows: *KPNA4* (forward CTGGACCAAACCTGGAGCAT; reverse AAGTGCTGCTGAGGTAAGCC), *FAK* (forward CTTCGGACAGCGTGAGAGAG; reverse AGTGTGCACAGCTCCATGAT), *PIK3CA* (forward GGTTTGGCCTGCTTTTGGAG; reverse CCATTGCCTCGACTTGCCTA), *AKT1* (forward CAGGATGTGGACCAACGTGA; reverse AAGGTGCGTTCGATGACAGT), *CD274* (forward GTTGTGGATCCAGTCACCTCT; reverse GGTCACTGCTTGTCCAGATGA), *GAPDH* (forward AACGGATTTGGTCGTATTG; reverse GGAAGATGGTGATGGGATT).

### Statistical Analysis

SPSS software version 20.0 was utilized for statistical analysis in our study. Wilcoxon matched-pairs signed rank test was performed to determine the expression differences of *KPNA4* between paired PDAC and normal pancreas samples in GSE15471, while Welch’s t test was performed in unpaired samples of GSE16515. The potential correlation between *KPNA4* expression and clinicopathologic characteristics in TCGA PDAC cohort was analyzed by Mann-Whitney test because the expression value disobeyed the Gaussian distribution. The association between KPNA4 histochemistry score and clinicopathologic parameters of our own PDAC clinical samples was analyzed by Pearson Chi-squared test. Cox regression analysis was carried out to evaluate the predictive value of KPNA4 in PDAC prognosis, and *KPNA4* expression was input as a categorical variable according to the median expression value. The factors with *p*-value < 0.2 in the univariate analysis were included in the subsequent multivariate analysis, where *p*-value < 0.05 was regarded to be statistically significant. Moreover, survival curves were generated by Graphpad Prism 7, and Log-rank test was implemented to determine the significant differences. Finally, correlation between *KPNA4* and FAK signaling-related genes in GSE15471 and GSE16515 was analyzed by the “stats” R package, and the “ggstatsplot” R package was used to visualize the result. The experimental data were presented as the mean ± standard deviation and each experiment was repeated three times per condition.

## Results

### KPNA4 Is Overexpressed in PDAC Samples

**Figure 1:**
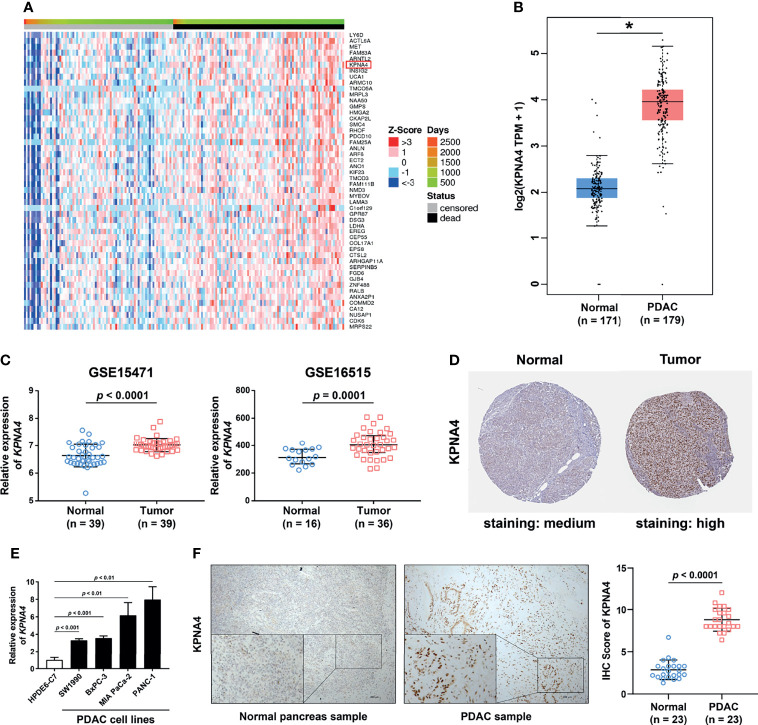
High expression of KPNA4 in PDAC. Heatmap of 50 genes which were most significantly positively associated with unfavorable survival in PDAC patients **(A)**. The mRNA expression of *KPNA4* was found to be significantly upregulated in PDAC tissues by using GEPIA **(B)** and GEO databases **(C)**. The protein level of KPNA4 was also remarkably increased in PDAC tissue by immunohistochemical images from the HPA database **(D)**. The upregulation of *KPNA4* in PDAC was verified in cell lines by qRT-PCR analysis **(E)**. The protein expression level of KPNA4 was validated in clinical samples by immunohistochemical staining. Scatter plot indicated the histochemistry score of KPNA4 in PDAC and matched normal tissues **(F)**. *p*-value < 0.05 was regarded statistically significant. *p < 0.05.

To excavate a novel immune-related biomarker with high prognostic value for PDAC patients, we first utilized the LinkedOmics database to obtain a list of genes with survival significance in TCGA PDAC, and ranked these genes according to their FDR value **(** **Figure 1A** and **Supplementary Table 2**). Remarkably, among the top six genes, *KPNA4* was the only one which has been reported to be associated with immune infiltration in malignancy while its roles in PDAC have not been studied yet (16). In addition, analysis *via* GEPIA database indicated that *KPNA4* was significantly upregulated in PDAC tissues (**Figure 1B**). Furthermore, *KPNA4* expression was significantly elevated in many other tumors including diffuse large B cell lymphoma (DLBC), esophageal carcinoma (ESCA), lung squamous cell carcinoma (LUSC) and stomach adenocarcinoma (STAD) (**Supplementary Figure 1A**). Therefore, we focused on KPNA4 for further analysis.

Next, we used Oncomine database to verify the expression of *KPNA4* in PDAC. As shown in **Supplementary Figure 1B**, although *KPNA4* showed discordant expression change in most cancer types, it was consistently upregulated in five cohorts of PDAC. Another cohort showed that *KPNA4* was also increased in pancreatic intraepithelial neoplasia, which is a precancerous lesion of pancreatic cancer **(** **Table 1**). In addition, two datasets from the GEO database (GSE15471 and GSE16515) consistently showed that the level of *KPNA4* was significantly upregulated in PDAC tissues (**Figure 1C**). To further verify the KPNA4 expression at the protein level, we compared the immunohistochemical staining of KPNA4 in normal pancreas and PDAC through the HPA database. The result again showed that KPNA4 expression in PDAC was markedly higher than that of normal pancreas, and KPNA4 showed obvious nuclear localization (**Figure 1D**).

**Table 1:** *KPNA4* expression change between PDAC and normal pancreas tissues (Oncomine).

Disease Type VS. Normal pancreas	Fold Change	*p*-Value	Reference
Pancreatic Ductal Adenocarcinoma	1.527	*1.87E-06*	(30)
Pancreatic Adenocarcinoma	1.349	*1.79E-04*	(31)
Pancreatic Carcinoma	1.761	*7.89E-04*	(32)
Pancreatic Intraepithelial Neoplasia	1.190	*1.80E-02*	(33)
Pancreatic Ductal Adenocarcinoma	1.504	*3.40E-02*	(34)
Pancreatic Carcinoma	1.297	*3.90E-02*	(35)

p-Value less than 0.05 are in italics.

To finally confirm, we measured the mRNA levels of *KPNA4* in cell lines by qRT-PCR, and then the protein levels in PDAC tissues by immunohistochemistry. qRT-PCR analysis revealed that *KPNA4* expression was remarkably elevated in PDAC cell lines SW1990, BxPC-3, MIA PaCa-2, and PANC-1 compared with that of human normal pancreatic ductal epithelial cell line HPDE6-C7 (**Figure 1E**). Immunohistochemistry further verified the upregulation of KPNA4 in our own clinical samples (**Figure 1F**). Altogether, these results suggest that KPNA4 may be a potential biomarker for PDAC.

### KPNA4 Is Closely Correlated With Clinicopathologic Characteristics of PDAC

**Figure 2:**
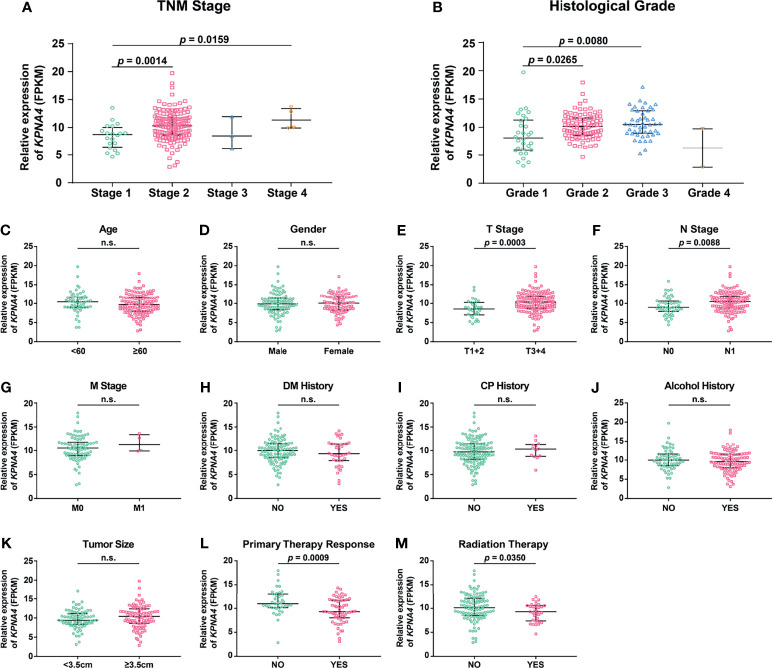
Association of *KPNA4* expression with clinicopathologic characteristics of PDAC patients. *KPNA4* expression in PDAC samples with different TNM stages **(A)** and histological grades **(B)**. TCGA PDAC samples with clinical data were used to analyze the correlation of *KPNA4* expression with other clinicopathologic parameters **(C–M)**. *p*-value < 0.05 was regarded statistically significant. n.s., no significance.

To investigate the relationship between *KPNA4* expression and clinicopathologic characteristics of PDAC patients, we initially analyzed the expression pattern of *KPNA4* in patients with different TNM stages and histological grades. Although the expression level of *KPNA4* was particular low in both Stage-3 and Grade-4 samples, probably due to the small sample sizes (only three Stage-3 samples and two Grade-4 samples), patients with higher stage and histological grade tended to express higher level of *KPNA4* (**Figures 2A, B**).

To further analyze the correlation of *KPNA4* with other clinicopathologic parameters, 173 PDAC samples with expression profiles and clinical data were downloaded from TCGA database, and then clinical correlation analysis were performed. The result showed that *KPNA4* expression had no correlation with age, gender, M stage, diabetes mellitus (DM) history, chronic pancreatitis (CP) history and tumor size (**Figures 2C, D, G–K**). However, tumors with higher T stage or N stage had a tendency to express higher level of *KPNA4* (**Figures 2E, F**), indicating that KPNA4 may promote tumor infiltration and lymph node metastasis. Notably, patients with higher *KPNA4* expression showed significantly poorer responses to primary therapy and radiotherapy (**Figures 2L, M**), suggesting that high *KPNA4*-expressed patients may be less sensitive to conventional therapies for PDAC. Additionally, the association between KPNA4 expression and clinicopathologic parameters of our own clinical samples is shown in **Supplementary Table 3**. The result showed that the high-KPNA4 group had a significantly higher ratio of patients in advanced TNM stage and histological grade, compared to the low-KPNA4 group. Altogether, these results suggest that KPNA4 expression is associated with tumor progression in PDAC patients.

### High-Expressed KPNA4 Is Independently Associated With a Poor Clinical Outcome in PDAC Patients

**Figure 3:**
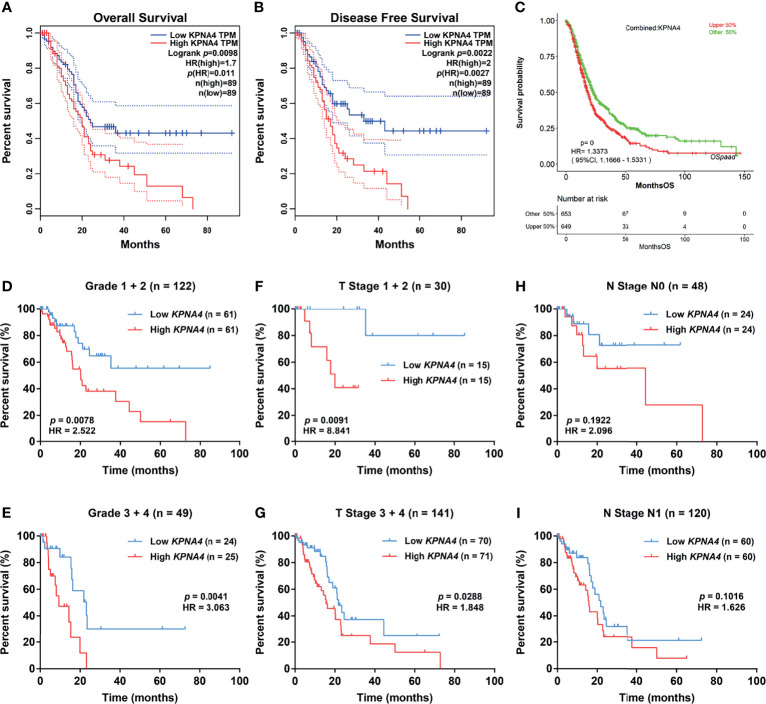
Survival analysis of *KPNA4* in PDAC. *KPNA4* expression was significantly associated with the overall survival and disease-free survival of PDAC patients **(A, B)**, which was further confirmed by OSpaad **(C)**. Stratified survival analysis revealed that high-expressed *KPNA4* was correlated with poor prognosis independent of histological grade **(D, E)** or T stage **(F, G)**, while *KPNA4* expression showed no significant association with survival when patients were stratified by N stage **(H, I)**. Log-rank *p-*value < 0.05 was regarded to be statistically significant.

Next, we evaluated the survival significance of *KPNA4* in PDAC patients. As we expected, patients with higher *KPNA4* expression showed significantly worse overall survival and disease-free survival (**Figures 3A, B**). Additionally, we verified the survival significance of *KPNA4* in a larger cohort *via* OSpaad, which is a web tool containing survival information of a total of 1319 PDAC cases from multiple databases. The result again showed that high *KPNA4* expression was associated with unfavorable survival outcome (**Figure 3C**). Subsequently, we carried out stratified survival analysis on 173 PDAC samples from TCGA database. The results further revealed that high *KPNA4* expression had a significant correlation with poor survival in patients regardless of histological grades (**Figures 3D, E**), indicating that the prognostic value of KPNA4 was independent of tumor malignancy. Similar results were observed when patients were stratified by T stage (**Figures 3F, G**). In addition, high *KPNA4* expression also indicated poor survival outcome when patients were stratified by N stage, although the result was not statistically significant (**Figures 3H, I**).

To further assess the prognostic value of KPNA4 in PDAC, we performed Cox regression analysis on 120 PDAC samples with complete clinical information and transcriptome data from TCGA database. Univariate analysis indicated that higher T stage, N stage and histological grade, larger tumor size and higher *KPNA4* expression were all correlated with worse survival (**Table 2**). While in multivariate analysis, only *KPNA4* expression was significantly correlated with the prognosis of PDAC patients. Taken together, KPNA4 holds great prognostic value among PDAC patients and thus could be a promising candidate of prognostic biomarker of PDAC.

**Table 2:** Univariate and multivariate Cox regression analyses of prognostic factors for overall survival in PDAC patients.

Risk factors	Univariate analysis			Multivariate analysis		
	HR	95% CI	p-Value	HR	95% CI	p-Value
Age (≥60 *vs*. <60)	1.144	0.604-2.166	0.679			
Gender (Female *vs*. Male)	0.800	0.458-1.400	0.435			
TNM stage (III+IV *vs*. I+II)	1.491	0.202-11.005	0.695			
T stage (T3+4 *vs*. T1+2)	3.389	1.328-8.650	*0.011*	2.192	0.757-6.344	0.148
N stage (N1 *vs*. N0)	2.512	1.277-4.939	*0.008*	1.282	0.558-2.946	0.558
Histological grade (G3+4 *vs*. G1+2)	1.545	0.877-2.721	*0.132*	1.333	0.732-2.430	0.347
DM history (YES *vs*. NO)	0.836	0.418-1.674	0.613			
CP history (YES *vs*. NO)	0.730	0.289-1.845	0.506			
Alcohol history (YES *vs*. NO)	1.307	0.714-2.393	0.385			
Tumor size (≥3.5cm *vs*. <3.5cm)	1.611	0.915-2.834	*0.098*	1.375	0.753-2.509	0.300
*KPNA4* expression	2.160	1.208-3.863	*0.009*	1.889	1.020-3.497	*0.043*

p-Value less than 0.2 in univariate analysis and 0.05 in multivariate analysis are in italics.

### Knockdown of *KPNA4* Inhibits PDAC Cell Proliferation and Migration

**Figure 4:**
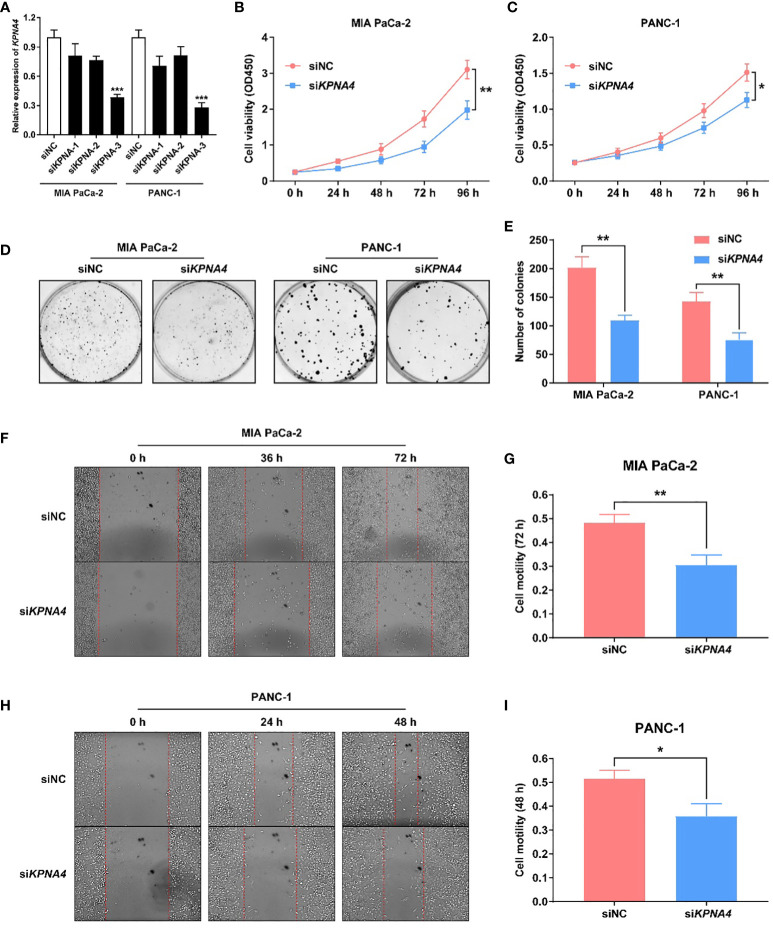
*KPNA4* knockdown inhibits proliferation and migration of PDAC cells. Knockdown efficiency of three siRNA on *KPNA4* was detected by qRT-PCR **(A)**. *KPNA4* knockdown suppressed cell proliferation of MIA PaCa-2 **(B)** and PANC-1 cells **(C)**. *KPNA4* knockdown inhibited the colony formation of MIA PaCa-2 and PANC-1 cells **(D, E)**. The inhibitory effect of *KPNA4* knockdown on MIA PaCa-2 **(F)** and PANC-1 **(H)** cell migration was evaluated by wound healing assay at different time points, and the migration index was shown in **(G, I)**. ^*^ *p* < 0.05, ^**^ *p* < 0.01, ^***^ *p* < 0.001.

To demonstrate the role of KPNA4 in the malignant behaviors of tumor cells, we first evaluated the transfection efficiency of si*KPNA4* by qRT-PCR. We used si*KPNA4*-3 for further studies because it had the most obvious inhibitory effect on *KPNA4* in MIA PaCa-2 and PANC-1 cells (**Figure 4A**). We performed CCK-8 assay to determine the effect of *KPNA4* knockdown on cell proliferation. The results indicated that *KPNA4* silencing significantly repressed MIA PaCa-2 and PANC-1 cell proliferation (**Figures 4B, C**). Colony formation assay also showed that *KPNA4* knockdown significantly inhibited the growth of these two PDAC cells (**Figures 4D, E**). Wound healing assay further revealed that after *KPNA4* knockdown, the migration ability of MIA PaCa-2 and PANC-1 cells decreased significantly compared with control cells (**Figures 4F–I**). In conclusion, these experimental data confirmed knockdown of *KPNA4* suppresses cell proliferation and migration in PDAC cells.

### KPNA4 Is Correlated With The Infiltration of Immunosuppressive Cells and T Cell Exhaustion in PDAC TME

**Figure 5:**
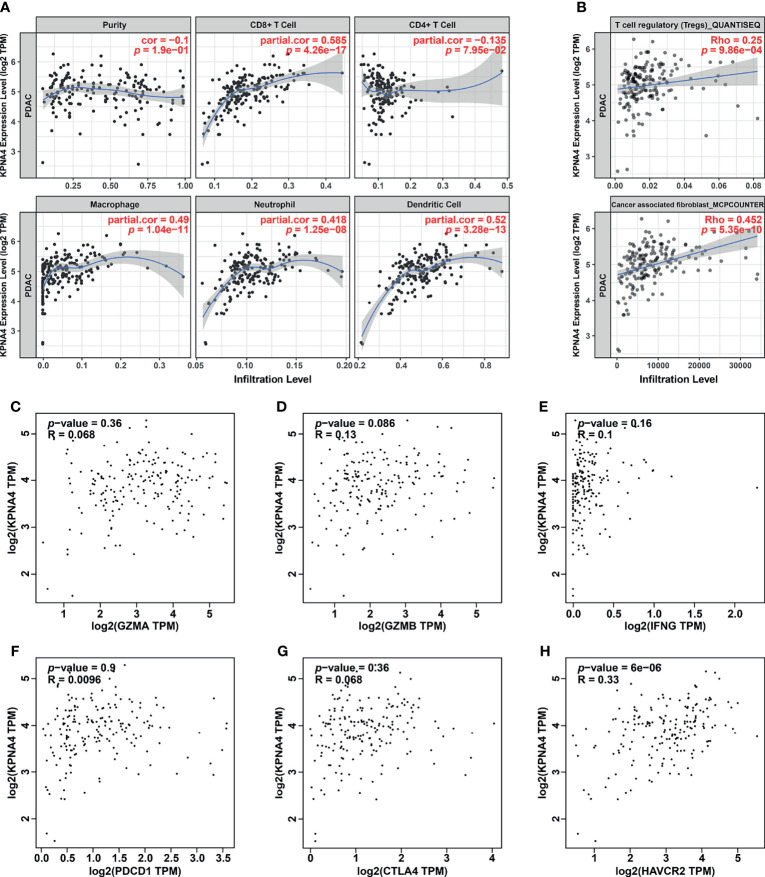
Immune infiltration analysis of KPNA4 in PDAC. The correlation between *KPNA4* expression and various immune cells infiltration in PDAC was analyzed using TIMER **(A)** as well as TIMER2.0 database **(B)**. The expression of *KPNA4* showed no significant relationship with cytotoxic CD8^+^ T cells markers (*GZMA*, *GZMB* and *IFNG*) **(C-E)**. There was no correlation between *KPNA4* expression and *PDCD1* and *CTLA4* **(F, G)**, while *KPNA4* was associated with *HAVCR2*, which is another marker of exhausted T cells **(H)**. *p*-value < 0.05 was considered to be statistically significant.

Given that immune cells in the TME have a central role in tumorigenesis and progression of PDAC (6), we next utilized TIMER database to analyze the association between *KPNA4* expression and immune infiltration in PDAC. As shown in **Figure 5A**, *KPNA4* expression had a significant positive correlation with the infiltration of CD8^+^ T cells, macrophages, neutrophils and dendritic cells (DCs). TIMER2.0 database further revealed that the expression of *KPNA4* was also positively correlated with the infiltration of regulatory T cells (Tregs) and cancer-associated fibroblasts (CAFs) (**Figure 5B**). While macrophages, neutrophils, DCs, Tregs and CAFs has been reported to have pro-tumorigenic effects in the TME (6, 36–39), the *KPNA4*-correlated infiltration of CD8^+^ T cells seemed to be inconsistent with the potential tumor-promoting role of *KPNA4*. Therefore, we further analyzed the relationship between the expression of *KPNA4* and cytotoxic CD8^+^ T cells markers *GZMA*, *GZMB* and *IFNG* in PDAC. Intriguingly, *KPNA4* showed no significant relationship with these effector genes (**Figures 5C–E**). Moreover, we explored the association between the expression of *KPNA4* and exhausted T cells markers. Although no significant correlation was found between expression levels of *KPNA4* and those of *PDCD1* (*PD-1*), *CTLA4*, *LAG3* or *TIGIT* (**Figures 5F, G** and **Supplementary Figures 2A, B**), we did find *KPNA4* was closely associated with *HAVCR2* (**Figure 5H**), which encodes the immune checkpoint receptor TIM3 and is tightly associated with poor prognosis of pancreatic cancer (40). In summary, these results suggest that high-expressed KPNA4 in PDAC may correlate with immunosuppressive cells infiltration and T cell exhaustion, thereby inhibiting anti-tumor immunity.

### Functional Analyses Associate KPNA4 With Focal Adhesion in PDAC

**Figure 6:**
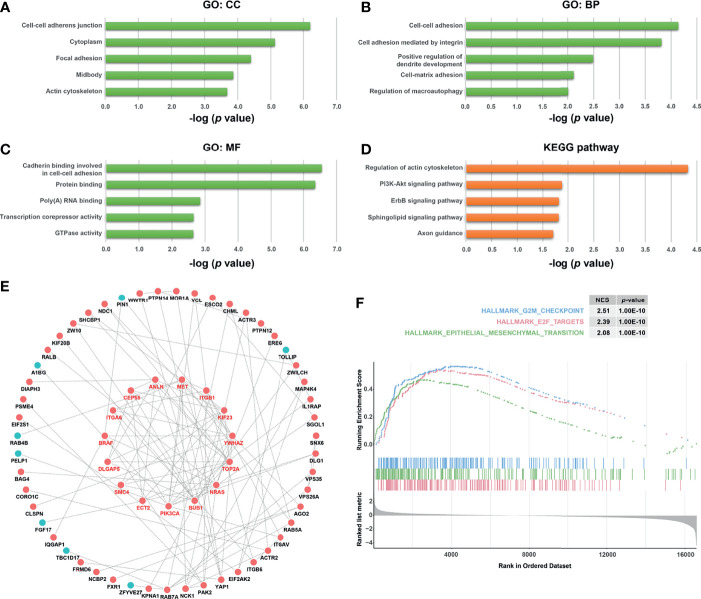
Functional analysis of KPNA4 by GO, KEGG pathway, PPI network and GSEA. GO analysis comprising cellular component (CC, **A**), biological process (BP, **B**) and molecular function (MF, **C**), and KEGG pathway analysis **(D)** were conducted on *KPNA4* co-expressed genes in TCGA PDAC. A PPI network was constructed by Cytoscape software based on these co-expressed genes, and the hub genes were calculated and presented in the form of circle in the middle of **(E)**. GSEA was carried out to compare the expression profiles between low-*KPNA4* and high-*KPNA4* samples in TCGA PDAC **(F)**. A *p*-value < 0.05 and FDR < 0.25 were considered to be a significantly enriched gene set.

To further explore the potential function and the underlying mechanism of KPNA4 in PDAC, we obtained *KPNA4* co-expressed genes in TCGA PDAC *via* LinkedOmics database (**Supplementary Table 4**). Next, the 200 positively and 200 negatively correlated genes with the highest correlation coefficient were selected as the *KPNA4* co-expressed gene set, and then functional enrichment analysis were carried out (**Supplementary Figures 3A, B**). The GO and KEGG pathway analysis revealed that KPNA4 was mainly involved in the regulation of cell-cell adhesion, cell-matrix adhesion and actin cytoskeleton remodeling (**Figures 6A–D**). Moreover, KEGG analysis showed that KPNA4 may also participate in regulating PI3K-Akt signaling pathway, which is a well-described therapeutic target for PDAC (**Figure 6D**) (41). Interestingly, focal adhesion is a type of integrin-mediated adhesive contact between cells and extracellular matrix, and its intracellular structure is tightly connected with the actin cytoskeleton, thereby regulating cell adhesion, cell motility and multiple cancer-related pathways including PI3K-Akt signaling (42, 43). Therefore, these results suggested that KPNA4 may regulate focal adhesion in PDAC. Furthermore, we analyzed *KPNA4* co-expressed genes by constructing a PPI network, and used Cytoscape software to identify the hub genes (**Figure 6E**). Noticeably, these hub genes were all positively correlated with *KPNA4*, and KEGG pathway analysis demonstrated that these genes were mostly enriched in PI3K-Akt signaling and focal adhesion (**Supplementary Figure 3C**), which further confirmed our previous assumption. Moreover, GSEA was performed for further investigation. Consistent with our finding of KPNA4 *in vitro*, the results revealed that genes involved in the G2/M checkpoint, genes encoding cell cycle related targets of E2F transcription factors, and genes related to EMT were notably enriched in high KPNA4-expressed samples (**Figure 6F** and **Supplementary Table 5**), suggesting that KPNA4 was also involved in these cancer-related pathways.

### KPNA4 Is Positively Related to FAK Signaling and PD-L1 Expression

**Figure 7:**
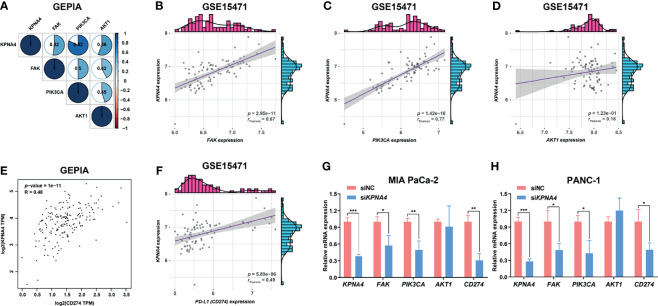
KPNA4 is positively related to FAK signaling and PD-L1 expression. Correlation analysis among the expression of *KPNA4*, *FAK*, *PIK3CA* and *AKT1* in TCGA PDAC **(A)**. In GSE15471, *KPNA4* expression was significantly correlated with *FAK* **(B)** and *PIK3CA* **(C)**, while no obvious association was observed between *KPNA4* and *AKT1* **(D)**. There was significant relationship between *KPNA4* expression and *PD-L1 (CD274)* in TCGA PDAC **(E)** and GSE15471 **(F)**. The regulatory effect of *KPNA4* knockdown on *FAK*, *PIK3CA*, *AKT1* and *CD274* expression in MIA PaCa-2 **(G)** and PANC-1 cells **(H)** by qRT-PCR analysis. ^*^ *p* < 0.05, ^**^ *p* < 0.01, ^***^ *p* < 0.001.

FAK overexpression has been considered as one of the key features of PDAC (44). Moreover, several studies have shown that PI3K pathway, which has been reported to promote tumor progression and metastasis, and restrict T cell recognition and clearance of tumor cells in pancreatic cancer (45, 46), is one of the main downstream signals of FAK (47). FAK-PI3K-Akt signaling is also important in non-neoplastic diseases, for example, this signaling pathway plays a key role in hepatic stellate cell proliferation and type I collagen expression in liver fibrosis (48). Given that KPNA4 may affect focal adhesion and PI3K-Akt signaling in our functional analysis, we next investigated the expression correlation of *KPNA4* with *FAK*, *PIK3CA* and *AKT1* in TCGA PDAC. Remarkably, *KPNA4* was found to be positively related to *FAK*, *PIK3CA* and *AKT1* with high correlation coefficient (**Figure 7A**). We further verified these correlations in two PDAC microarrays from the GEO database. The results showed that *KPNA4* was highly positively correlated with *FAK* and *PIK3CA* in GSE15471 (**Figures 7B, C**), although there was no significant correlation with *AKT1* (**Figure 7D**). Also, the expression of *KPNA4* showed a positive correlation with all these genes in GSE16515 (**Supplementary Figures 4A–C**). These results further indicated that KPNA4 may involve in FAK signaling in PDAC. Recent studies have demonstrated that FAK can mediate downstream of αVβ3 integrin to positively regulate the expression of PD-L1 in tumor cells (49). Thus, we wondered whether the expression of KPNA4 was related to PD-L1. Remarkably, analysis indicated that *KPNA4* had a significant and positive correlation with *CD274* (encoding PD-L1) (**Figure 7E**), and the relationship was further verified in GSE15471 and GSE16515 datasets (**Figure 7F** and **Supplementary Figure 4D**). To experimentally validate these findings, we knocked down *KPNA4* by small interfering RNA (si*KPNA4*) in MIA PaCa-2 and PANC-1 pancreatic cancer cell lines with high *KPNA4* expression (**Figures 7G, H**). Consistently, the expression levels of *FAK*, *PIK3CA* and *CD274* were significantly downregulated after *KPNA4* knockdown, whereas *AKT1* expression had nothing to do with *KPNA4*. Overall, our results suggested that KPNA4 may promote FAK signaling and PD-L1 expression in PDAC cells, which could be responsible for the KPNA4-dependent malignant behaviors of cancer cells and immunosuppressive TME in PDAC patients.

## Discussion

PDAC is one of the most lethal malignancies and is often diagnosed at an advanced stage with high mortality rates (50). Therefore, it is necessary to find accurate biomarkers for the early detection and prognostic prediction of PDAC. Recent studies have reported several potential biomarkers for PDAC. For example, COL11A1 was considered to be a prognostic marker of PDAC and was significantly related to tumor immune infiltration (51). Additionally, S100A16 was discovered as an important biomarker for the diagnosis and prognosis of PDAC (52).

Studies have shown that the KPNA family as nuclear transport factors can mediate the translocation of several transcription factors to the nucleus, and is closely associated with a variety of cancers including PDAC (53). For instance, high expressions of KPNA2 and KPNA7 have been found to be significantly correlated with poor prognosis in PDAC (54, 55). In the present study, we focused on KPNA4, which is a member of the KPNA family (11). According to a recent study, high-expressed KPNA4 in hepatocellular carcinoma (HCC) can indicate poor survival outcome, supporting KPNA4 as an independent predictor of HCC prognosis (16). However, the prognostic value of KPNA4 in other cancers has yet to be reported. To this end, we first analyzed the expression of KPNA4 in PDAC *via* bioinformatics methods. The results showed that the mRNA and protein levels of KPNA4 were significantly increased in PDAC compared with that in normal pancreas tissues, which was further verified in cell lines and our own clinical samples. Further clinical correlation analysis demonstrated that patients with advanced stage and high histological grade tended to express higher level of *KPNA4*, and *KPNA4* expression was also associated with tumor infiltration and lymph node metastasis. Moreover, patients with high-expressed *KPNA4* had a tendency to show poorer response to therapy. These results were highly consistent with the survival significance of KPNA4 in PDAC. Stratified survival analysis and Cox regression analysis further revealed that high *KPNA4* expression could independently predict unfavorable prognosis. Thus, all these results above suggest that KPNA4 can act as a potential prognostic biomarker for PDAC patients.

It has been reported that only a minority of pancreatic cancers are immunologically active, and immune cells in the TME, especially immunosuppressive cells, are thought to play a vital role in this process (6, 9). Previous research revealed a positive relationship between KPNA4 and immune cell infiltration in HCC, suggesting that KPNA4 may participate in regulating TME (16). However, whether KPNA4 relates to immune infiltration in other cancers has not been elucidated yet. In our work, immune infiltration analysis demonstrated that *KPNA4* expression was significantly and positively correlated with the infiltration of macrophage, neutrophil, DCs, Tregs and CAFs in PDAC TME. Tumor-associated macrophages suppress the function of nature killer cells and T cells by expressing non-classical MHC class I molecules and ligands of immune checkpoint receptors, thereby promoting tumor growth and invasion (6). High levels of intratumoral neutrophil infiltration are considered to be significantly correlated with poor prognosis of multiple cancers (37). The infiltration of intratumoral Tregs increases with the progression of PDAC, and also has significant relationship with unfavorable survival outcome (36). Moreover, CAFs, which are the main producers of extracellular matrix proteins, are considered to be implicated in many tumor-promoting processes (38). Therefore, KPNA4 may mediate the infiltration of these immunosuppressive immune cells, thereby promoting cancer progression. Moreover, our study revealed that high *KPNA4* expression was significantly positively correlated with the infiltration of CD8^+^ T cells, which have potential tumoricidal activity. Nevertheless, there was no relationship between the expression of *KPNA4* and cytotoxic CD8^+^ T cells markers (*GZMA*, *GZMB* and *IFNG*). Further analysis revealed the correlation between KPNA4 and TIM3 (encoded by *HAVCR2*), which is a marker of exhausted T cells and has been reported to be tightly associated with poor survival outcome in PDAC (40). These results collectively demonstrate that although KPNA4 is potentially related to CD8^+^ T cells infiltration in PDAC, it may shape an immunosuppressive TME and promote T cell exhaustion, thus inhibiting cytotoxic T cells and anti-tumor response.

The indirect interaction between FAK and integrin at focal adhesion canonically mediates FAK signaling, which has been proven to regulate motility, invasion and survival of cancer cells, thereby promoting cancer growth and metastasis (17). Overexpression of FAK is regarded as a key feature of PDAC and many studies have demonstrated the anti-tumor effect of FAK inhibitors on human pancreatic cancer cells (44, 56). Intriguingly, functional enrichment analysis and PPI network in our study illustrate that *KPNA4* co-expressed genes in PDAC are mainly involved in focal adhesion and PI3K-Akt pathways, which has been reported to be the downstream pathway of FAK signaling in PDAC (57). Correlation analysis also revealed that *KPNA4*, *FAK*, *PIK3CA*, and *AKT1* showed a highly positive correlation with each other. Moreover, *in vitro* experiment further indicated that *KPNA4* knockdown had a negative regulatory effect on the expression of *FAK* and *PIK3CA*. Consistently, our loss-of-function experiments revealed that *KPNA4* silencing suppressed proliferation and migration of PDAC cells, confirming its tumor-supporting roles in pancreatic cancer. Hereby we postulate that KPNA4 may play a tumor-promoting role *via* FAK signaling. Further studies are necessary to unravel the underlying mechanisms.

Increasing evidence has shown that FAK can exert immunomodulatory roles in cancers. FAK signaling create the immunosuppressive TME by regulating the expression of chemokines such as CXCL12 and CCL5, enabling tumors to escape anti-tumor immunity (58). More importantly, FAK was found to function downstream of αVβ3 integrin to positively regulate the interferon signaling, and then promote the nuclear translocation of transcription factor STAT1/2, thereby activating PD-L1 expression and promoting tumor immune escape (49). Our study illustrated that *KPNA4* was positively related to *PD-L1* expression in multiple PDAC cohorts, and *KPNA4* silencing significantly decreased *PD-L1* expression in PDAC cell lines. Notably, studies have elucidated that KPNA family can mediate the nuclear transport of a variety of transcription factors including STAT1/2 (59). Although our results showed that *KPNA4* knockdown suppressed *FAK* expression *in vitro*, these previous researches lead to our assumption that KPNA4 may also act downstream of FAK signaling and affect the expression of PD-L1. Furthermore, although PDAC is regarded as an immunologically cold tumor type, studies have revealed that patients with PD-L1^-^/CD8^high^ subtype are still associated with better prognosis, indicating that T cell infiltration in the absence of immunoinhibiting PD-L1 can still exert its obvious tumoricidal roles (60). Therefore, considering that KPNA4 may participate in shaping the immunosuppressive TME, KPNA4-targeted strategy is expected to reverse the tumor-promoting microenvironment and enhance anti-tumor immunity.

Still, our study has some limitations. Majority of the data used in our work came from online databases, thus a large-scale study is needed for further validation. Additionally, proliferation and migration promoting functions of KPNA4 *in vitro* require to be verified by *in vivo* xenograft tumor model and metastasis model. Further *in vitro* and *in vivo* experiments are needed to explore the specific mechanism of KPNA4 in PDAC and its immunosuppressive TME.

## Conclusions

In conclusion, our study explored the expression and prognostic value of KPNA4 in PDAC patients, revealing that KPNA4 can be used as an independent prognostic biomarker for PDAC. KPNA4 exerts tumor-promoting roles in PDAC cells and is associated with the immunosuppressive TME, which may be partly mediated by FAK signaling and PD-L1 expression. On this basis, further experimental researches are needed to uncover the exact mechanisms, so as to provide a new therapeutic target for therapy of pancreatic cancer.

## Data Availability Statement

The original contributions presented in the study are included in the article/**Supplementary Material**. Further inquiries can be directed to the corresponding author.

## Ethics Statement

The studies involving human participants were reviewed and approved by Ethics Committee of Shanghai General Hospital, Shanghai Jiaotong University School of Medicine. The patients/participants provided their written informed consent to participate in this study.

## Author Contributions

J-PB and C-LX contributed equally to design the study, obtain the data, and write the manuscript. G-YH reviewed the manuscript and supervised the study. BL, Z-KW, and JS collected the clinical samples and analyzed the results. P-LS and QP assisted in statistical analysis. All authors contributed to the article and approved the submitted version.

## Funding

This study was sponsored by National Natural Science Foundation of China (81970556 and 82170652).

## Conflict of Interest

The authors declare that the research was conducted in the absence of any commercial or financial relationships that could be construed as a potential conflict of interest.

## Publisher’s Note

All claims expressed in this article are solely those of the authors and do not necessarily represent those of their affiliated organizations, or those of the publisher, the editors and the reviewers. Any product that may be evaluated in this article, or claim that may be made by its manufacturer, is not guaranteed or endorsed by the publisher.
