# Corrigendum: Systematic characterization of the clinical relevance of KPNA4 in pancreatic ductal adenocarcinoma

**DOI:** 10.3389/fonc.2023.1282120

**Published:** 2023-09-11

**Authors:** Jingpiao Bao, Chaoliang Xu, Bin Li, Zengkai Wu, Jie Shen, Pengli Song, Qi Peng, Guoyong Hu

**Affiliations:** ^1^ Department of Gastroenterology, Shanghai General Hospital, Shanghai Jiao Tong University School of Medicine, Shanghai, China; ^2^ Shanghai Key Laboratory of Pancreatic Disease, Institute of Pancreatic Disease, Shanghai Jiao Tong University School of Medicine, Shanghai, China; ^3^ Laboratory of Cancer Genomics and Biology, Department of Urology, Shanghai General Hospital, Shanghai Jiao Tong University School of Medicine, Shanghai, China

**Keywords:** karyopherin subunit alpha 4, pancreatic ductal adenocarcinoma, prognostic biomarker, tumor microenvironment, FAK signaling

**Error in Figure**

In the published article, there was an error in **Figures 4F** and **4H** as published. The group names were erroneously interchanged. The corrected **Figures 4F** and **4H** and its caption “**Figure 4**. KPNA4 knockdown inhibits proliferation and migration of PDAC cells” appear below.

**Figure 4:**
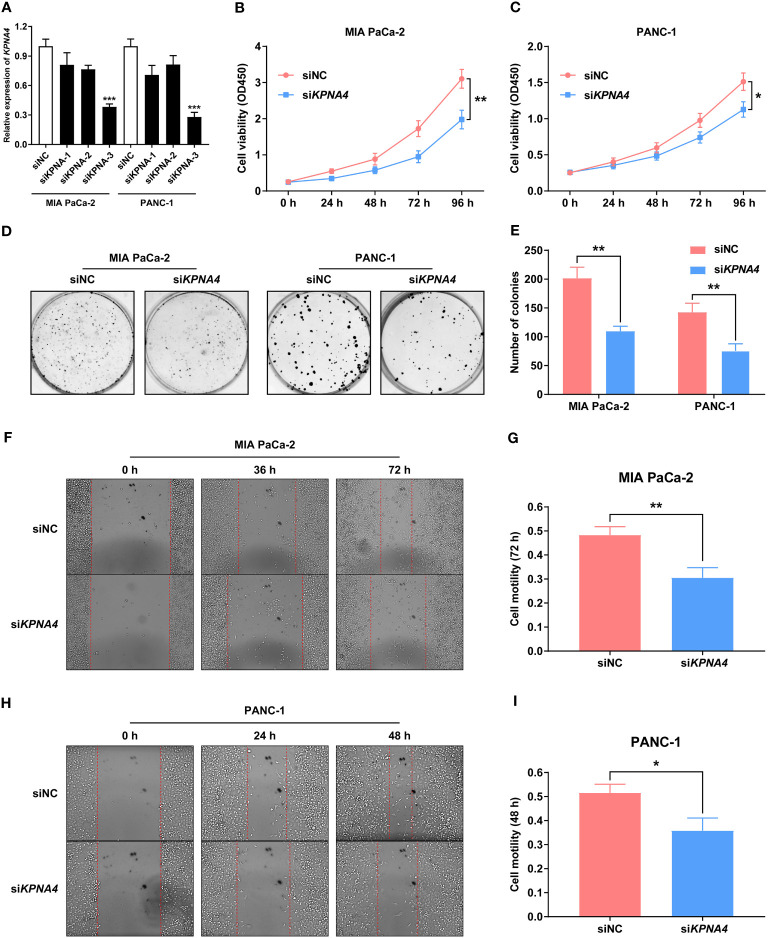
*KPNA4* knockdown inhibits proliferation and migration of PDAC cells. Knockdown efficiency of three siRNA on *KPNA4* was detected by qRT-PCR **(A)**. *KPNA4* knockdown suppressed cell proliferation of MIA PaCa-2 **(B)** and PANC-1 cells **(C)**. *KPNA4* knockdown inhibited the colony formation of MIA PaCa-2 and PANC-1 cells **(D, E)**. The inhibitory effect of *KPNA4* knockdown on MIA PaCa-2 **(F)** and PANC-1 **(H)** cell migration was evaluated by wound healing assay at different time points, and the migration index was shown in **(G, I)**. ^*^ *p* < 0.05, ^**^ *p* < 0.01, ^***^ *p* < 0.001.

We apologize for this error and state that this does not change the scientific conclusions of the article in any way. The original article has been updated.

