# Supplementary material for: Systematic Characterization of the Clinical Relevance of KPNA4 in Pancreatic Ductal Adenocarcinoma
Source: Front Oncol. 2022 Mar 29;12:834728. doi: 10.3389/fonc.2022.834728 (PMC9002131; doi:10.3389/fonc.2022.834728)
Supplement: Supplementary file 1 [file DataSheet_1.pdf]

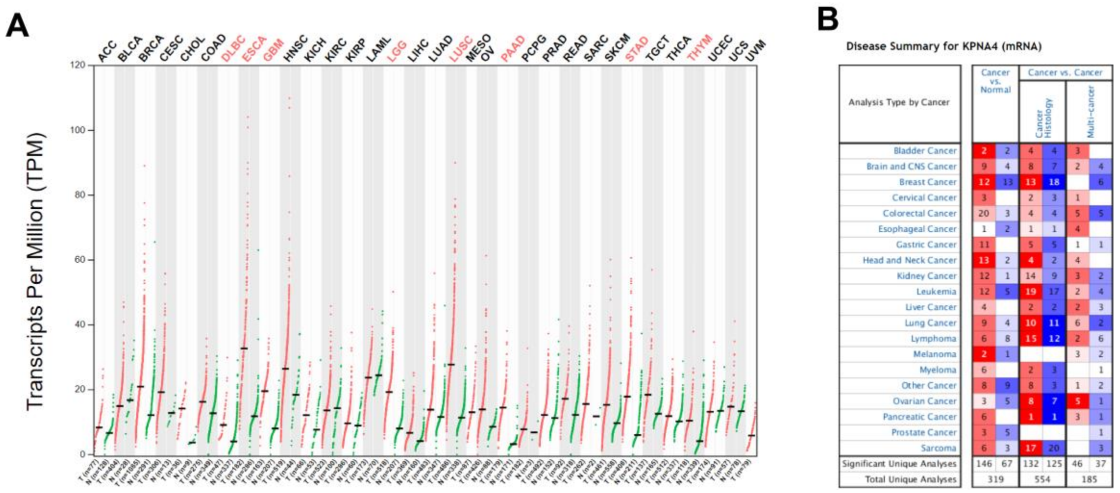

Figure S1. *KPNA4* was found to be markedly upregulated in several cancers by GEPIA database (**A**). The mRNA expression of *KPNA4* was found to be significantly upregulated in different PDAC cohorts by using Oncomine database (**B**).

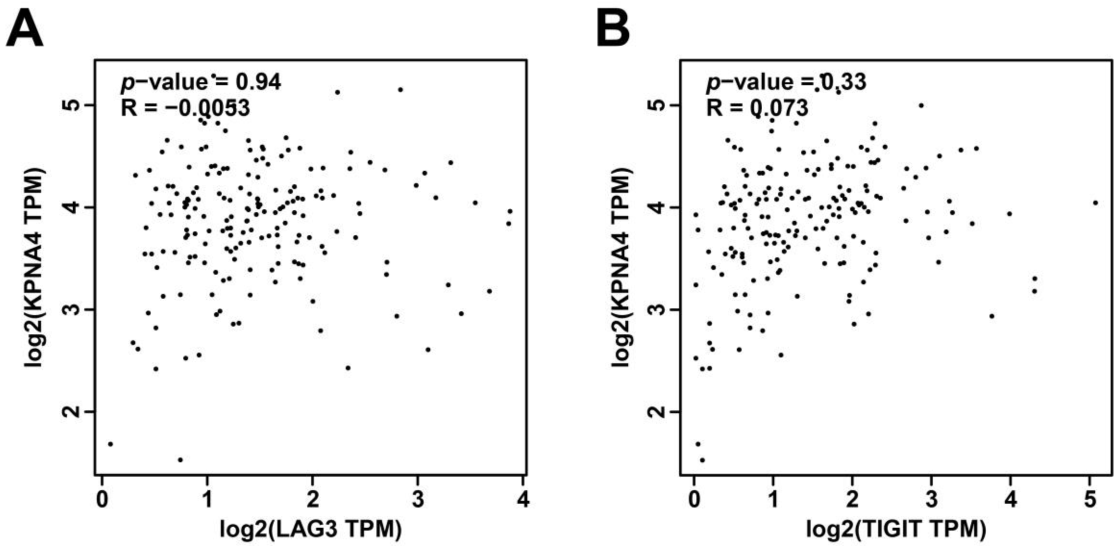

Figure S2. *KPNA4* showed no significant correlation with the exhausted T cells markers *LAG3* (**A**) and *TIGIT* (**B**). *p*-value < 0.05 was considered to be statistically significant.

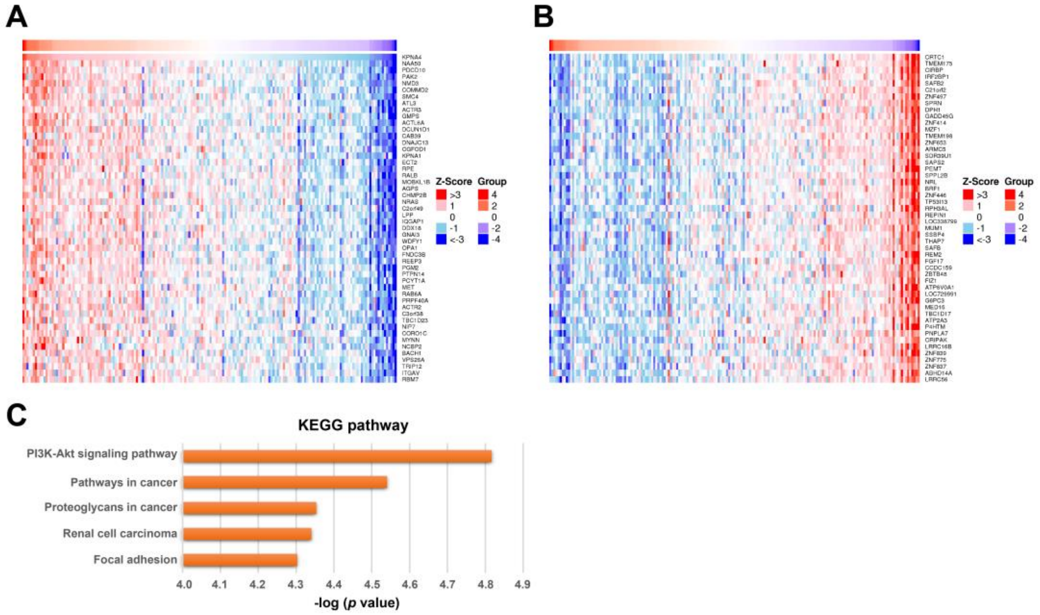

Figure S3. Heatmap of genes positively and negatively co-expressed with *KPNA4* in TCGA PDAC (**A**, **B**). KEGG pathway analysis on the hub genes of *KPNA4* co-expressed genes PPI network (**C**).

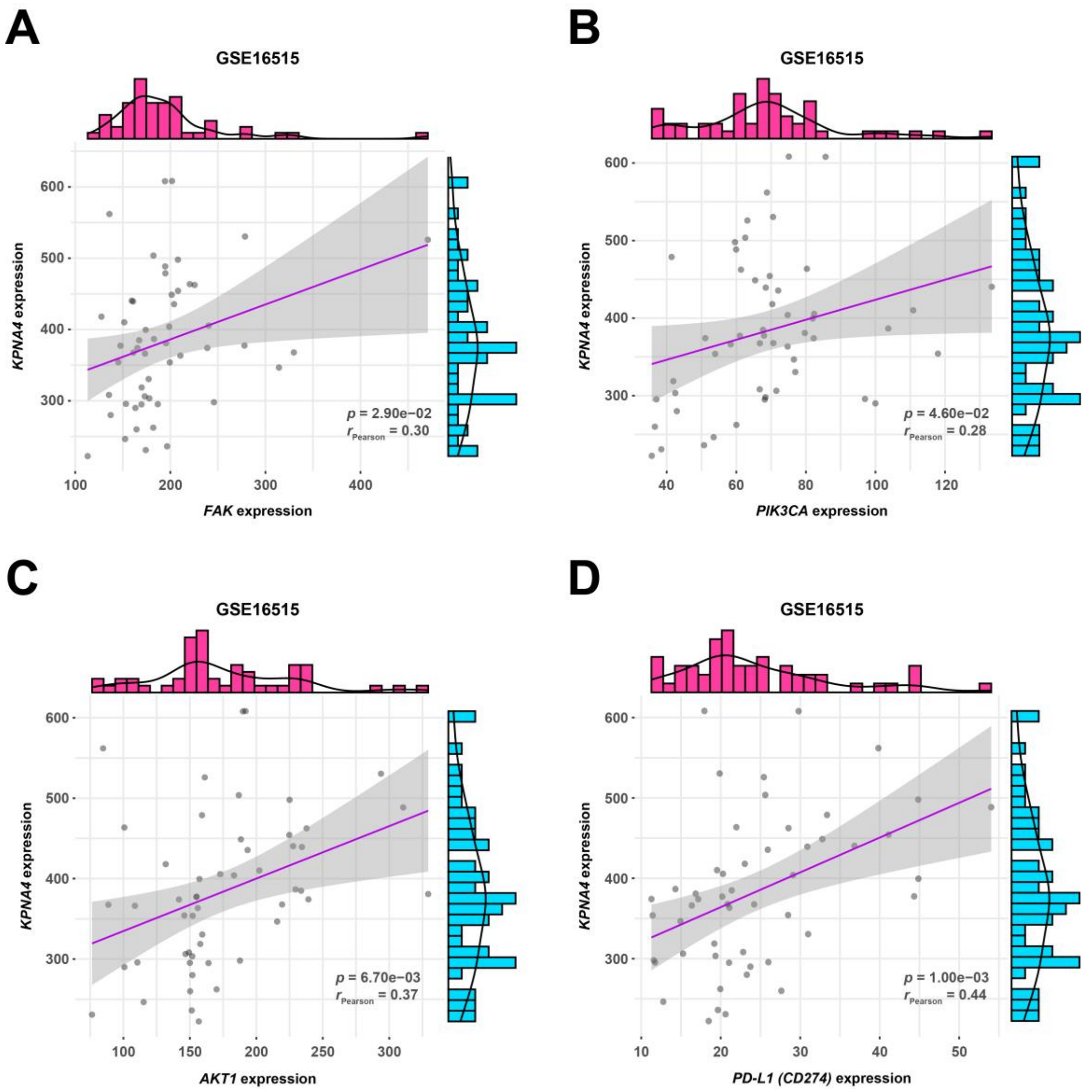

Figure S4. *KPNA4* showed significant positive correlations with *FAK* (**A**), as well as its downstream effector *PIK3CA* (**B**) and *AKT1* (**C**) in GSE16515. There was significant relationship between *KPNA4* expression and *PD-L1 (CD274)* in GSE16515 (**D**). *p*-value < 0.05 was considered as statistically significant.
